# Difference between papillary and follicular thyroid carcinoma outcomes: an experience from Egyptian institution

**DOI:** 10.7497/j.issn.2095-3941.2015.0005

**Published:** 2015-03

**Authors:** Engy M. Aboelnaga, Rehab Allah Ahmed

**Affiliations:** 1 Clinical Oncology and Nuclear Medicine Department; 2 Pathology Department, Faculty of Medicine, Mansoura University, Mansoura 35511, Egypt

**Keywords:** Thyroid cancer, differentiated thyroid, papillary, follicular, Egypt

## Abstract

**Objective:**

Differentiated thyroid carcinomas (DTCs) are classified into papillary thyroid carcinoma (PTC) and follicular thyroid carcinoma (FTC). DTCs are analyzed as a single group in clinical studies that investigated the prognostic factors and prognosis of these malignancies. However, the biological behaviors of these carcinomas significantly differ. In the present study, we aimed to detect differences in the outcomes between PTC and FTC in Mansoura University Hospital in Egypt.

**Methods:**

A total of 558 patients with histologically proven thyroid carcinomas from January 2003 to December 2012 were retrospectively enrolled. The clinical and pathological data of patients were reviewed.

**Results:**

Large primary tumor size, lymph node involvement, extrathyroid extension, and distant metastasis were significant poor prognostic factors for overall survival (OS) in old PTC patients. Cox hazard analysis showed that the patient’s age, extra thyroid extension, and distant metastasis were the only independent prognostic factors. In FTC patients, only the distant metastasis and degree of tumor invasion were significant poor prognostic factors in OS univariate analysis. However, these factors were nonsignificant in multivariate analysis. The 10-year OS rates were 97% and 89% for PTC and FTC, respectively (*P*=0.003). The 10-year disease-free survival (DFS) rates were 77.2% in PTC *vs*. 65% in FTC (*P*=0.179).

**Conclusion:**

The significant prognostic factors vary between the two types of DTCs. Therefore, PTC and FTC patients need to be analyzed and reported independently. PTC survival is widely and significantly affected by age, extrathyroid extension, and distant metastasis. By contrast, these factors were nonsignificant in FTC, which showed poorer survival than PTC.

## Introduction

Differentiated thyroid carcinoma (DTC) originates from epithelial follicular cell and considered as a major form of thyroid carcinoma. DTC includes two histological types, namely, papillary thyroid carcinoma (PTC) and follicular thyroid carcinoma (FTC)^1^.

DTC is mostly indolent and a clinically nonsignificant disease that is found incidentally with good prognosis in most cases and a high 10-year survival rate (90%-95%)^2^.

A thyroid nodule is the first symptom of thyroid cancer and of particular concern when found in patients younger than 20 years old, with greater potential for malignant presentation in such cases. Pain in the anterior region of the neck and changes in voice may occur as late symptoms because of the involvement of the recurrent laryngeal nerve^3^.

Approximately 80%-85% of all thyroid malignancies are PTCs. In addition to the classic form of PTCs, several morphological variants, usually classified as biologically aggressive, have been identified: diffuse sclerosing, tall cell, columnar cell, solid/trabecular, and insular variants^4^. The aggressive behavior of PTC is attributed to its invasion into extrathyroidal tissues and extensive vascular invasion^5^. However, recent studies have shown that some of these variants (sclerosing and tall cell) are not related to poor outcome *per se*, but rather tumor prognosis is dependent on the presence of aggressive features, such as extracapsular and vascular invasion, larger tumor size, and the presence of distant metastasis (DM)^6,7^. FTC is the second most frequent subtype and accounts for approximately 10%-15% of all thyroid cancers. Hürthle cell thyroid carcinoma is a variant of FTC that accounts for 3% of thyroid malignancies and is characterized by less sensitivity to iodine therapy^8,9^.

DTCs are analyzed as a single group in clinical studies that investigate the prognostic factors and prognosis of patients. However, the biological behaviors of the two types of DTC significantly vary. PTC metastasizes frequently to the regional lymph nodes and may show a high incidence of significant extrathyroid extension to adjacent organs. By contrast, FTC more frequently metastasizes to more distant organs, such as the lung, bone, and brain, than PTC^10^.

PTC and FTC are often treated similarly despite their numerous biological differences. The most effective management of thyroid cancers is the surgical removal of the thyroid gland (thyroidectomy), followed by radioactive iodine (RAI) ablation and thyroid replacement therapy for TSH suppression. Chemotherapy or radiotherapy has a limited role only in cases with DM or advanced cancer stage^11^.

Unlike most cancers, recurrence of DTC does not necessarily correlate with increased risk of mortality. This phenomenon is particularly demonstrated in young patients who have higher rates of local recurrence, but have low mortality risk. Several clinical features enable initial risk stratification, including patient age, size of the primary tumor, histology, gross extra-thyroidal extension, completeness of resection, involvement of the cervical lymph nodes, or DM^12^.

In the present study, we aimed to distinguish the outcomes of prognosis between PTC and FTC in a single institution (Mansoura University Hospital in Egypt).

## Patients and methods

### Patient data

A total of 558 patients, with histologically proven thyroid carcinoma from January 2003 to December 2012, were retrospectively included in this study. This study was approved by the ethics committee at Mansoura University Hospital.

The data were collected from the archives of patients in the clinical oncology and nuclear medicine departments, as well as those in the pathology department. The data included the general characteristics of patients and tumor: age, sex, primary tumor size, the number of lymph node metastasis, multifocality of the primary lesion, extrathyroid extension, subtypes of papillary and follicular thyroid carcinoma, TNM stage based on TNM staging of the American Joint Committee on Cancer, and the development of DM^13^. Extrathyroid extension was defined according to the TNM criteria as follows: any tumor with minimal extrathyroid extension as extension to sternothyroid muscle or perithyroid soft tissues (PT_3_); tumor of any size that extends beyond the thyroid capsule and invades subcutaneous soft tissues, larynx, trachea, esophagus, or recurrent laryngeal nerve (PT_4a_); or tumor that invades the prevertebral fascia or encases carotid artery (PT_4b_). Patients with focal poorly differentiated carcinoma, focal undifferentiated carcinoma, or DM at the time of diagnosis were excluded from this study.

Treatment modalities, which included the type of surgical resection and either hemi-thyroidectomy, near, or subtotal thyroidectomy with lymph node dissection, were performed in positive lymph node cases. Redo surgery (completion thyroidectomy) was performed in cases with postoperative large residual tumor. Postoperative treatment, which included radioiodine ablation ^131^I (80-120 mCi) in patients who underwent at least a near-total thyroidectomy and thyroxine suppression therapy, was also recorded. Chemotherapy was used in some cases of metastatic or non-resectable DTC, and radiotherapy was used as palliative treatment for metastatic or unresectable cases.

Follow-up data of patients were retrieved from the archive of the Clinical Oncology and Nuclear Medicine Department, and the survival data, including the overall survival (OS) and disease-free survival (DFS), were collected.

DFS was calculated from the date of surgery until the date of recurrence (either local or distant), and OS was calculated from the date of diagnosis until the date of death of the patient (regardless of the cause of death).

### Statistical analysis

Data were analyzed using Statistical Package for Social Sciences Version 16 (SPSS Inc., Chicago, IL, USA). Qualitative data were presented as number and percentage. Survival curves were estimated by the Kaplan-Meier method with log rank test to assess significance. Multivariate Cox proportional hazard regression models were used to evaluate any independent prognostic effect of the variables with 95% confidence interval (CI). A *P*-value of <0.05 was considered significant.

## Results

In the present retrospective study, 558 patients were enrolled, and their data were analyzed with median follow-up period of 52 months (range: 12-120 months). A total of 477 patients (85.5%) were diagnosed with PTC, whereas FTC was found in 81 (14.5%) patients.

Other patients’ characteristics are shown in **Table 1**.

**Table 1:** Clinicopathological features of all thyroid carcinoma patients

Characteristics	PTC (*n*=477)			FTC (*n*=81)	
	*n*	%		*n*	%
Gender					
Male	145	30.4		23	28.4
Female	332	69.6		58	71.6
Male: female ratio	01:02.2			01:02.5	
Patients’ age (years)					
<45	284	59.5		37	45.7
≥45	193	40.5		44	54.3
Median in years (range)	40 (8-71)			40 (21-72)	
Primary tumor size (cm)					
≤1	57	11.9		2	2.5
>1 and ≤2	110	23.1		7	8.6
>2 and ≤4	223	46.8		37	45.7
>4	87	18.2		35	43.2
Lymph node status					
N_0_	302	63.3		75	92.6
N_1_	175	36.7		6	7.4
Development of DM					
No	456	95.6		59	72.8
Positive	21	4.4		22	27.2
Extrathyroid extension					
No	444	93.1		77	95.1
Probable	33	6.9		4	4.9
Multifocality					
No	440	92.2		78	96.3
Yes	37	7.8		3	3.7
Subtypes of PTC/FTC					
Classic/classic	331	69.4		58	71.6
Follicular/Hürthle cell carcinoma	133	27.9		23	28.4
Others*	13	2.7		–	–
Degree of invasion					
Minimally invasive	–	–		48	59.3
Widely invasive				33	40.7

*, Including encapsulated tall and columnar cell variants. DM, distant metastasis; PTC, papillary thyroid carcinoma; FTC, follicular thyroid carcinoma.

### Prognostic features of PTC

Our results showed that old age, large primary tumor size, lymph node stage, extrathyroid extension, and DM were significant poor prognostic factors for PTC on the univariate analysis of OS (**Table 2**). Multivariate COX hazard analysis showed that patient age (HR =5.94; 95% CI, 1.237-28.650; *P*=0.026), extrathyroid extension (HR =3.527; 95% CI, 1.070-11.626; *P*=0.038), and DM (HR =12.968; 95% CI, 3.924-42.854; *P*<0.001) were the only independent prognostic factors of OS. Results from the univariate analysis of the various prognostic factors in the PTC patients’ series in relation to DFS using log rank test are summarized in **Table 2**. Our results showed that male gender, old age, large primary tumor size, lymph node metastasis, extrathyroid extension, and multifocality were significant poor prognostic factors, but the multivariate COX hazard analysis showed that tumor size (HR =3.972; 95% CI, 1.237-15.650; *P*=0.023) and lymph node stage (HR =5.434; 95% CI, 1.245-26.530; *P*=0.014) were the only independent prognostic factors (**Table 3**).

**Table 2:** Prognostic variables in PTC in relation to OS and DFS

Characteristics	OS (%)	*P*	DFS (%)	*P*
Gender		0.668		0.001
Male	96.7		67.9	
Female	97.7		82.5	
Patients’ age (years)		<0.001		<0.001
<45	98.8		85.8	
≥45	94.2		66.5	
Primary tumor size (cm)		0.002		<0.001
≤1	100		91.5	
>1 and ≤2	100		89.2	
>2 and ≤4	96.8		79.9	
>4	90.3		40.3	
Lymph node status		<0.001		<0.001
N_0_	100		94.9	
N_1_	91.8		49.1	
Development of DM		<0.001	–	–
No	98.6			
Positive	63.2			
Extrathyroid extension		<0.001		<0.001
No	98		82	
Probable	84.4		28	
Multifocality		0.94		0.002
No	97		79.9	
Yes	94.1		55.9	
Subtypes		0.15		0.642
Classic	98		98	
Follicular	94.4		94.4	
Others*	100		100	

*, Including encapsulated tall and columnar cell variants. PTC, papillary thyroid carcinoma; OS, overall survival; DFS, disease-free survival; DM, distant metastasis.

**Table 3:** COX proportional hazard analysis of the predictors of DFS in the PTC series

Variable	*P*	HR	95% CI	
			Lower	Upper
Gender	0.356	0.607	0.21	1.752
Patients’ age	0.791	0.884	0.354	2.204
Tumor size	0.033	1.892	1.051	3.404
LN stage	0.002	4.975	1.845	13.411
Multifocality	0.644	1.313	0.414	4.159
Extrathyroid extension	0.075	2.72	0.904	8.18

PTC, papillary thyroid carcinoma; DFS, disease-free survival.

We studied the differences in survival between the various types of PTC in terms of the OS and DFS, and no significant relationship was found [OS (LR =3.788, *P*=0.150) or DFS (LR =0.798, *P*=0.671)].

### Prognostic features of FTC

We found that the only factors that significantly affected the OS of FTC patients were DM and degree of tumor invasion (**Table 4**). However, none of these remained independent in the multivariate analysis. The factors that affected the DFS of FTC on univariate analysis are summarized in **Table 4**. The results showed that tumor size, extrathyroid extension, and degree of tumor invasion were the only significant poor prognostic factors in FTC patients. Only extrathyroid extension (HR =10.956; 95% CI, 2.724-39.864; *P*<0.001) and degree of tumor invasion (HR =5.547; 95% CI, 1.684-27.754; *P*=0.025) remained independent in the multivariate analysis in our patients’ series (**Table 5**). However, the various types of FTC did not exhibit any significant effect on OS (*P*=0.097) or DFS (*P*=0.410).

**Table 4:** Prognostic variables in FTC in relation to OS and DFS

Characteristics	OS (%)	*P*	DFS (%)	*P*
Gender		0.822		0.675
Male	88.5		57.1	
Female	90.5		67.3	
Patients’ age (years)		0.253		0.102
<45	94.1		73.5	
≥45	84.6		56.4	
Primary tumor size (cm)		0.432		0.017
≤1	100		50	
>1 and ≤2	80		80	
>2 and ≤4	94.1		76.5	
>4	84.4		50	
Lymph node status		0.504		0.789
N_0_	88.1		64.1	
N_1_	100		75	
Development of DM		<0.001	–	–
No	98.1			
Positive	66.7			
Extrathyroid extension		0.16		0.002
No	90		67.1	
Yes	66.7		0	
Multifocality		0.94		0.055
No	97		47	
Yes	94.1		0	
Subtypes		0.097		0.41
Classic	92.6		66.7	
Hürthle cell carcinoma	78.9		57.9	
Degree of invasion		0.004		<0.001
Minimally invasive	97.5		82.5	
Widely invasive	78.6		39.3	

FTC, follicular thyroid carcinoma; OS, overall survival; DFS, disease-free survival; DM, distant metastasis.

**Table 5:** COX proportional hazard analysis of the predictors of DFS in the FTC series

Variable	*P*	HR	95% CI	
			Lower	Upper
Tumor size, cm	0.244	0.576	0.227	1.458
Extrathyroid extension	0.021	4.937	1.279	19.057
Degree of invasion	<0.001	13.118	3.364	51.144

DFS, disease-free survival; FTC, follicular thyroid carcinoma.

### Treatment

Total or near total thyroidectomy was performed in 97.1% of our patients. Hemithyroidectomy was performed in 2.9%, and redo surgery was performed in 57 patients (10.2%). Chemotherapy or radiotherapy was applied alone or in combination with other lines in 11 and 16 patients, respectively. RAI was applied in 552 (98.9%) patients. This information is reported as descriptive results only.

### Survival

**Figure 1:**
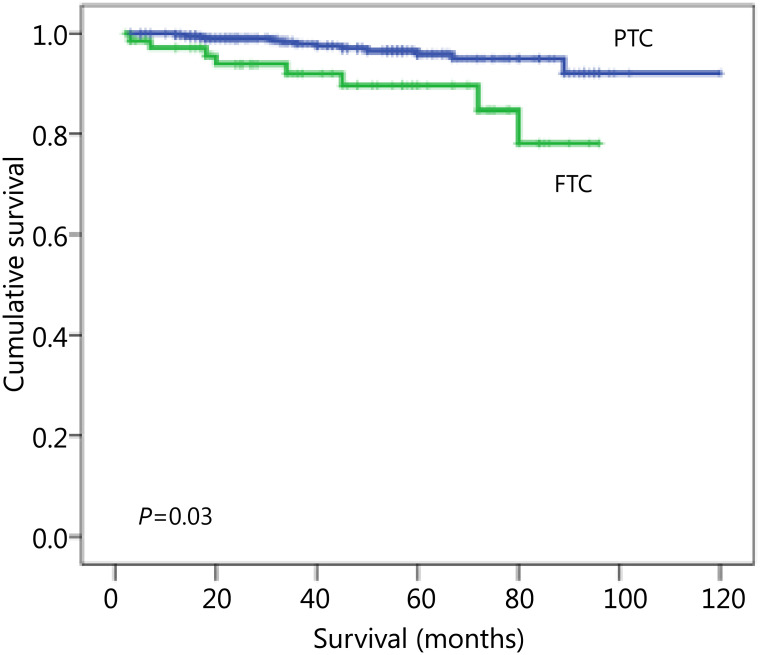
Kaplan-Meier plot of OS for PTC and FTC. OS, overall survival; PTC, papillary thyroid carcinoma; FTC, follicular thyroid carcinoma.

The 10-year OS rates were 97% and 89% for PTC and FTC, respectively, with a statistically significant difference (*P*=0.003) (**Figure 1**). The 10-year DFS rates were 77.2% in PTC *vs*. 65% in FTC, without statistically significant difference (*P*=0.179).

## Discussion

PTC and FTC are generally indolent diseases and show good prognosis if treated appropriately. These DTCs are studied as a single group in investigative studies on the prognosis of thyroid carcinoma, in spite of their distinct biological behavior. Therefore, prognostic factors and prognosis of PTC and FTC patients should be analyzed independently^14^. In the present study, we evaluated the factors that affected the prognosis of PTC and FTC in Egypt and compared them with other reports. Our results showed that the significant prognostic factors differed between the two types of DTC. However, some studies could not distinguish the PTC and FTC outcomes^10–15^, and other reports have considered a significantly poorer prognosis for FTC^16,17^, which is consistent with our report.

The age of PTC patients in our study ranged from 8 to 71 years old, with a median of 43 years old for PTC. By contrast, the age of FTC patients, ranged from 21 to 72 years old with a median of 43 years old. Other studies have reported an age range from 25 to 65 years old with the median age at diagnosis of 49 years old^15^. These results agree with previous studies demonstrating that PTC and FTC can occur in children (accounting for 80% and 10%-20%, respectively)^16^.

Female patients were more common than males in our study with a ratio of 2.28:1 in PTC and 2.5:1 for FTC, which agrees with the results of another report^15^.

Ages older than 45 years old affected the OS and DFS of PTC patients on the univariate analysis. However, this factor was not an independent prognostic factor, contrary to previous results^18^. Other studies have reported that its prognostic effect on DFS was weak^19^. However, some studies have stated that ages 45 years old or older are a significant prognostic factor for FTC on univariate analysis (which is not consistent with our results), but are not an independent prognostic factor^20^.

A previous study has demonstrated that gender only has a weak prognostic significance in DTC, with women showing better survival advantage than men. This result was attributed to a younger age distribution among women, and if age was also considered, gender would not exhibit prognostic significance^21^. However, other studies have reported that male gender was an independent prognostic factor for PTC and FTC^18,22^. By contrast, our study showed the sex of a patient had no effect on DFS and OS for both PTC and FTC. The difference in the results can be attributed to the difference in the numbers of cases.

In our study, multifocality had no effect on the OS of PTC and FTC patients. However, multifocality significantly influenced the DFS of PTC on the univariate analysis, but not on the multivariate analysis, which agrees with previous results^19^. Passler *et al*. stated that multifocality significantly affects the outcome of both PTC and FTC patients^18^. One study has reported that PTC with lymph node metastasis is more frequently detected in multiple than in solitary microcarcinomas and suggested that multiplicity reflects the aggressive behavior of PTC to some extent^23^.

Tumor size of larger than 4 cm significantly affected OS and DFS of PTC patients, which is consistent with other studies^18,22^. Therefore, careful and extensive surgery, as well as postoperative follow-up, is recommended for PTC tumor sizes larger than 4 cm even in the absence of other high-risk features. In FTC, the size of larger than 4 cm had marginal significance for DFS in FTC, but not for cause-specific survival (CSS) on multivariate analysis^20^.

Lymph node metastasis was not an independent prognostic factor in our series, contrary to previous reports, which determined that this is an independent prognostic factor in PTC^18,19^. Other studies have found that pathological node metastasis increases the rate of carcinoma recurrence^23^.

We found that lymph node metastasis did not affect the outcome of FTC, which could be explained by the low possibility of FTC to metastasize to the regional lymph node, and lymph-node dissection is rarely performed during surgery for patients suspected of FTC. Thus, evaluating the prognostic value of this factor is important. Previous studies have found that FTC with pathological node metastasis show an aggressive pathology, e.g., widely invasive carcinoma, and extensive lymph-node dissection is recommended for these FTC cases^20^. Passler *et al*.^18^ found that involvement of cervical lymph nodes is statistically significant in univariate analysis only in patients >45 years of age.

In our result and in those of other studies, the variant of PTC did not significantly affect survival^24^. The tall cell variant has been reported to be typical with aggressive behavior and independently affects DFS and CSS of PTC patients, as shown by multivariate analysis^25^. The difference in the results could be attributed to the very small number of this variant in our study.

The survival in Hürthle cell carcinoma was lower than the classic variant in the present study, but no statistical significance was found. Previous studies have found that most cases of oxyphilic cell variant were RAI refractory with worse prognosis than conventional FTC^18^. Moreover, the prognosis of oxyphilic cell variant is reportedly similar to or even better than that of conventional FTC^20^.

Similar to the reported results, the extrathyroid extension in our study was an independent prognostic factor for OS and DFS in PTC and DFS in FTC^18,19^. A rare event of extrathyroid extension in FTC had been reported and accounted for only 2% of patients and predicted an adverse prognosis^20^.

The widely invasive FTC is more likely to recur and exhibit poorer prognosis than the minimally invasive FTC. Therefore, whether an FTC is widely or minimally invasive should be diagnosed during pathological examination to predict patient prognosis and select a subsequent therapy^20^. In the present series, OS and DFS in widely invasive FTC were significantly lower than those of patients with minimally invasive FTC, which agrees with other studies. Thus, completion of total thyroidectomy followed by RAI ablation for widely invasive FTC patients is advised^18^.

Although the incidence of DM in PTC is lower than that in FTC, PTC can metastasize not only to regional lymph nodes, but also to distant organs, such as the lung, bone, and brain. This study showed that the development of DM was an independent prognostic factor of PTC and FTC, as stated in previous reports^18^. DM at surgery is one of the most important prognostic factors for CSS of patients^19^.

Total or near-total thyroidectomy is primarily recommended to reduce the risk of recurrence, thereby improving survival^26^. Bilimoria *et al*.^27^ demonstrated that total thyroidectomy results in lower recurrence rates and improves survival for PTC compared with lobectomy. However, less radical surgery for all low-risk patients is preferred in some studies^28^.

Postoperative treatment consisted of radioactive ^131^I (80-120 mCi) in patients who underwent at least a near-total thyroidectomy and thyroxin suppression therapy. This treatment was used to ablate residual normal thyroid tissue and adjuvant therapy of subclinically or clinically apparent residual disease. Several studies have suggested a 50% 10-year reduction in recurrence risk and decrease in mortality rate and risk of metastasis^29,30^. Chemotherapy was used in some cases of metastatic, non-resectable, or iodine non-responder-differentiated thyroid cancer. However, this modality may result in rare complete remission and uncommon long-term responses^30^. Several single chemotherapeutic agents are used in thyroid cancer. Additionally, this treatment has no benefit when used in combination therapy to improve the overall response, but may instead increase toxicity^30^.

In conclusion, the significant prognostic factors differ between the two types of DTC. Therefore, PTC and FTC patients should be analyzed and reported independently.

PTC prognosis is widely and significantly affected by the age and extrathyroid extension compared with FTC. Additionally, survival of FTC patients is significantly poorer than that of PTC patients.

## References

[1] Pacini F, Schlumberger M, Dralle H, Elisei R, Smit JW, Wiersinga W (2006). European consensus for the management of patients with differentiated thyroid carcinoma of the follicular epithelium. Eur J Endocrinol.

[2] Hu MI, Vassilopoulou-Sellin R, Lustig R, Lamont JP, Pazdur R, Wagman LD, Camphausen KA, Hoskins WJ (2008). Cancer Management: A Multidisciplinary Approach.

[3] Sawka AM, Thephamongkhol K, Brouwers M, Thabane L, Browman G, Gerstein HC (2004). Clinical review 170: A systematic review and metaanalysis of the effectiveness of radioactive iodine remnant ablation for well-differentiated thyroid cancer. J Clin Endocrinol Metab.

[4] Livolsi VA, Albores-Saavedra J, Asa SL, DeLellis RA, Lloyd R, LiVolsi VA, Eng C (2004). Pathology and Genetics of Tumors of the Endocrine Organs.

[5] Volante M, Landolfi S, Chiusa L, Palestini N, Motta M, Codegone A (2004). Poorly differentiated carcinomas of the thyroid with trabecular, insular, and solid patterns: a clinicopathologic study of 183 patients. Cancer.

[6] Regalbuto C, Malandrino P, Tumminia A, Le Moli R, Vigneri R, Pezzino V (2011). A diffuse sclerosing variant of papillary thyroid carcinoma: clinical and pathologic features and outcomes of 34 consecutive cases. Thyroid.

[7] Silver CE, Owen RP, Rodrigo JP, Rinaldo A, Devaney KO, Ferlito A (2011). Aggressive variants of papillary thyroid carcinoma. Head Neck.

[8] Lo CY, Chan WF, Lam KY, Wan KY (2005). Follicular thyroid carcinoma: the role of histology and staging systems in predicting survival. Ann Surg.

[9] D’Avanzo A, Treseler P, Ituarte PH, Wong M, Streja L, Greenspan FS (2004). Follicular thyroid carcinoma: histology and prognosis. Cancer.

[10] Cushing SL, Palme CE, Audet N, Eski S, Walfish PG, Freeman JL (2004). Prognostic factors in well-differentiated thyroid carcinoma. Laryngoscope.

[11] Hundahl SA, Fleming ID, Fremgen AM, Menck HR (1998). A National Cancer Data Base report on 53,856 cases of thyroid carcinoma treated in the U.S., 1985-1995.. Cancer.

[12] Carter WB, Tourtelot JB, Savell JG, Lilienfeld H (2011). New treatments and shifting paradigms in differentiated thyroid cancer management. Cancer Control.

[13] Edge SB, Byrd DR, Compton CC, Fritz AG, Greene FL, Trotti A (2012). AJCC Cancer Staging Manual.

[14] Ito Y, Miyauchi A (2012). Prognostic factors of papillary and follicular carcinomas in Japan based on data of kuma hospital. J Thyroid Res.

[15] Altekruse SF, Kosary CL, Krapcho M, Neyman N, Aminou R, Waldron W (2010). SEER Cancer Statistics Review, 1975-2007, National Cancer Institute.

[16] Williams D (2008). Radiation carcinogenesis: lessons from Chernobyl. Oncogene.

[17] Chen GG, Vlantis AC, Zeng Q, van Hasselt CA (2008). Regulation of cell growth by estrogen signaling and potential targets in thyroid cancer. Curr Cancer Drug Targets.

[18] Passler C, Scheuba C, Prager G, Kaczirek K, Kaserer K, Zettinig G (2004). Prognostic factors of papillary and follicular thyroid cancer: differences in an iodine-replete endemic goiter region. Endocr Relat Cancer.

[19] Ito Y, Ichihara K, Masuoka H, Fukushima M, Inoue H, Kihara M (2010). Establishment of an intraoperative staging system (iStage) by improving UICC TNM classification system for papillary thyroid carcinoma. World J Surg.

[20] It Y, Hirokawa M, Higashiyama T, Takamura Y, Miya A, Kobayashi K (2007). Prognosis and prognostic factors of follicular carcinoma in Japan: importance of postoperative pathological examination. World J Surg.

[21] Kuriakose MA, Hicks WL, Loree TR, Yee H (2001). Risk group-based management of differentiated thyroid carcinoma. J R Coll Surg Edinb.

[22] Ito Y, Miyauchi A (2009). Prognostic factors and therapeutic strategies for differentiated carcinomas of the thyroid. Endocr J.

[23] Ito Y, Tomoda C, Uruno T, Takamura Y, Miya A, Kobayashi K (2004). Papillary microcarcinoma of the thyroid: how should it be treated?. World J Surg.

[24] Hagag P, Hod N, Kummer E, Cohenpour M, Horne T, Weiss M (2006). Follicular variant of papillary thyroid carcinoma: clinical-pathological characterization and long-term follow-up. Cancer J.

[25] Ito Y, Hirokawa M, Fukushima M, Inoue H, Yabuta T, Uruno T (2008). Prevalence and prognostic significance of poor differentiation and tall cell variant in papillary carcinoma in Japan. World J Surg.

[26] Duren M, Yavuz N, Bukey Y, Ozyegin MA, Gundogdu S, Açbay O (2000). Impact of initial surgical treatment on survival of patients with differentiated thyroid cancer: experience of an endocrine surgery center in an iodine-deficient region. World J Surg.

[27] Bilimoria KY, Bentrem DJ, Ko CY, Stewart AK, Winchester DP, Talamonti MS (2007). Extent of surgery affects survival for papillary thyroid cancer. Ann Surg.

[28] Udelsman R, Chen H (1999). The current management of thyroid cancer. Adv Surg.

[29] Mazzaferri EL, Jhiang SM (1994). Long-term impact of initial surgical and medical therapy on papillary and follicular thyroid cancer. Am J Med.

[30] Gottlieb JA, Hill CS (1974). Chemotherapy of thyroid cancer with adriamycin. Experience with 30 patients. N Engl J Med.

